# Experimental exposure of *Burkholderia pseudomallei* crude culture filtrate upregulates PD-1 on T lymphocytes

**DOI:** 10.1099/acmi.0.000110

**Published:** 2020-02-14

**Authors:** Nivedita Menon, Vanitha Mariappan, Kumutha M. Vellasamy, Chandramathi Samudi, Jia-Xiang See, P. Sankar Ganesh, Alireza Saeidi, Jamuna Vadivelu, Esaki Muthu Shankar

**Affiliations:** ^1^​ Department of Medical Microbiology, Faculty of Medicine, University of Malaya, Lembah Pantai, Kuala Lumpur, Malaysia; ^2^​ Department of Life Sciences, Central University of Tamil Nadu, Thiruvarur 610 005, Tamilnadu, India; ^3^​ Department of Pediatrics, Emory University, Atlanta, GA, USA; ^‡^​ Present address: Department of Medical Microbiology, Faculty of Medicine, University of Malaya, Lembah Pantai, Kuala Lumpur, Malaysia; ^§^​ Present address: Department of Life Sciences, Central University of Tamil Nadu, Tamilnadu, Thiruvarur, India

**Keywords:** *Burkholderia pseudomallei*, PD-1, culture filtrate, immune responses

## Abstract

*
Burkholderia pseudomallei* is the causative agent for melioidosis. Because of its intracellular nature, the bacterium is capable of replicating within a plethora of eukaryotic cell lines. *B. pseudomallei* can remain dormant within host cells without symptoms for years, causing recrudescent infections. Here, we investigated the pathogenesis mechanism behind the suppression of T cell responses by *B. pseudomallei *. Peripheral blood mononuclear cells (1×10^6^ cells/well) isolated by Ficoll Paque (Sigma-Aldrich) density gradient centrifugation were incubated with optimized concentrations of bacterial crude culture filtrate antigens (CFAs) (10 ug ml^−1^) and heat-killed bacteria [1 : 10 multiplicity of infection (m.o.i.)]. Following incubation, cells were investigated for surface expression of coinhibitory molecules by flow cytometry. We found that *B. pseudomallei* induced the upregulation of programmed death 1 (PD-1), a molecule responsible for T cell exhaustion, on T cells *in vitro* following exposure to crude CFAs of *B. pseudomallei *. This upregulation of PD-1 probably contributes to poor immune surveillance and disease pathogenesis.

## INTRODUCTION

*Burkholderia pseudomallei *causes melioidosis, a deadly infectious disease of humans and animals leading to significant mortality rates that is often reported from parts of Southeast Asia and northern Australia [1–3]. Over the past few decades, it has become a major focus of global concern [4, 5]. The intrinsic ability of *B. pseudomallei* to resist various antibiotics makes it less vulnerable to antibiotic therapy, meaning there is an urgent need to develop an effective vaccine against melioidosis [6]. *B. pseudomallei *, a category B bioterrorism agent [7], is acquired via inhalation of aerosolized bacteria, ingestion of contaminated water or by cutaneous inoculation [8]. Clinical manifestations include localized infections, with the lungs being the most commonly affected organ, followed by the liver and spleen [9]. Other protean manifestations, such as pneumonia and septic shock, are associated with high mortality rates. *B. pseudomallei* is a facultative intracellular pathogen that can invade, multiply and thrive within phagocytic and non-phagocytic cells [10–12]. This ability allows them to remain quiescent in the host, resulting in the recurrence of symptoms for several years after infection [13–15].

A recent study on a murine macrophage-like cell line showed that *B. pseudomallei* can combat host proteases in the phagosome by releasing a serine protease inhibitor called ecotin [16]. This, together with the type 3 secretion system (T3SS), allows the bacteria to escape phagosomal killing by macrophages [17, 18]. Upon escape, the bacteria spread to surrounding cells and form multinucleated giant cells [19]. Another mechanism adopted by *B. pseudomallei* to escape phagocytosis is the induction of caspase-1-dependant cell death in macrophages [20]. However, stimulation of macrophages with IFN-γ notably enhances antibacterial ability, limiting the intracellular survival of *B. pseudomallei* [21–23]. This is because macrophage-mediated killing requires optimal levels of IFN-γ via T cell and NK cell activation and inadequate levels would allow *B. pseudomallei* to escape innate immune responses [24, 25]. Since *B. pseudomallei* can evade macrophages, the more versatile cell-mediated immunity (CMI) involving the expansion of T cells could be essential. The fact that clinical melioidosis samples show reduced T cell counts indicates that *B. pseudomallei* could be impairing T cell activation to evade immune surveillance [26, 27]. Nonetheless, the exact mechanism behind T cell suppression during melioidosis is poorly understood.

T cells are activated by dendritic cells (DCs) and the engagement of co-signalling molecules on both the cells helps to positively (co-stimulation) and negatively (co-inhibition) regulate T cell activation. PD-1 receptor is found on the surface of CD4+ and CD8+ T cells and is upregulated within 24–72 h of TCR stimulation [28]. Further engagement of PD-1 with its ligand(s) PD-L1/L2 inhibits T cell proliferation and cytokine secretion [29]. In an acute infection, such an inhibitory signal would limit the number of effector T cells during T cell expansion [30], whereas during chronic infection, due to persistent exposure to antigens, this interaction renders the T cells unresponsive, leading to T cell exhaustion [31]. Increasing evidence suggests that PD-1 has a role in the inhibition of effector T cell responses in persistent *Mycobacterium tuberculosis* [32] and persistent viral infections [33–35], which we demonstrated in a murine model of persistent *B. pseudomallei* infection [36]. Upregulation of PD-L1 on polymorphonuclear neutrophils (PMNs) of melioidosis patients has been shown to impair T cell functions [37]. Nonetheless, there is no evidence to date regarding PD-1 upregulation on T cells following exposure to crude culture filtrate antigens of *B. pseudomallei *. Here, we investigated the pathogenesis mechanisms behind the suppression of T cell responses by *B. pseudomallei *.

## METHODS

### Ethical approval

All experiments involving humans were performed in accordance with the relevant guidelines and regulations and under examination by the Medical Ethics Committee (MEC) of University Malaya Medical Centre (UMMC), Kuala Lumpur, Malaysia (ref. no. 1017.23), and were conducted per the guidelines of the International Conference on Harmonization Guidelines and the Declaration of Helsinki. All individuals involved in the study were over 18 years of age and provided informed consent to participate in the study.

### Blood samples

Blood samples (10 ml) from healthy subjects at UMMC, Malaysia were collected in sodium heparin BD Vacutainers (BD Biosciences, Franklin Lakes, NJ, USA). Peripheral blood mononuclear cells were isolated within 8 h of phlebotomy by Ficoll Paque (Sigma-Aldrich) density gradient centrifugation. Cells were counted using the trypan blue exclusion method. PBMCs were seeded in six-well tissue culture plates at 1×10^6^ cells/well.

### Bacterial strains

Three bacterial strains were used: a clinical isolate of *B. pseudomallei* (THE), obtained from the spleen of a patient admitted to UMMC; a virulent environmental isolate, *Burkholderia thailandensis* (ATCC); and the K96243 strain (mouse spleen). The clinical isolate was identified as *B. pseudomallei* through its ability to grow on Ashdown agar, and also by molecular confirmation using groEL-specific primer for genus detection and mprA-specific primer for species characterization. We also performed substrate utilization tests using the API20NE test according to the manufacturer’s instructions.

Twenty-four hour growth curves, colony-forming units (c.f.u.) ml^−1^ and multiplicity of infection (m.o.i.) were determined for all strains (data not shown). Heat inactivation of bacteria was performed as described previously [38–40]. Briefly, all strains were grown in Luria–Bertani (LB) broth and incubated overnight (37 °C at 200 r.p.m.) in the shaking incubator. The OD at 590 nm for each tube was checked the following day and all cultured tubes were adjusted to the same OD using phosphate-buffered saline (PBS). The samples were serially diluted and plated to determine the number of viable cells. The cells were harvested and washed twice using PBS. The bacterial suspension was heat-inactivated (HI) at 80 °C in 5 mM PBS (pH 7.3) in a water bath. The bacterial cells were harvested by centrifugation and resuspended in PBS and stored at 4 °C until use.

### Extraction of culture filtrate antigens

Crude CFA was extracted as described previously [41]. Briefly, the strains were grown in LB broth. The culture was centrifuged at 20 000 ***g*** for 40 min. The supernatant was harvested and filtered through a 0.22 µM filter (Sartorius, Goettingen, Germany). The filtered supernatant was concentrated 50-fold using a Pierce Concentrator 9K (Thermo Scientific, USA). The protein content was estimated by Bradford assay against a bovine serum albumin (Biowest, USA) standard [42]. The preparations were stored at −20 °C until use. PBMCs (1×10^6^ cells) were seeded into six-well plates and incubated with optimized concentrations of CFA (10 ug ml^−1^) and heat-killed bacteria (1 : 10 m.o.i.) for 36 h. Antigen-unexposed mock cells were used as a negative control, and cells stimulated with phytohaemagglutin (PHA) (1.5 % v/v) were used as a positive control.

### Flow cytometry

Following incubation with antigens, the cells were investigated for surface expression of co-inhibitory receptors. All antibodies were pretitrated to determine appropriate working concentrations. Cells were stained with Fixable Viability Stain (FVS510, BD Biosciences; clone R35-95) and incubated for 20 min. Monoclonal antibodies directed against CD3 (BD Biosciences clone UCHT1), CD4 (BD Biosciences clone SK7), CD8 (BD Biosciences clone SK1) and PD-1 (BD Biosciences clone MIH4), CTLA-4 (BD Biosciences clone BNI3) and TIM-3 (R and D Systems clone #344823) were added and incubated for 30 min. The samples were washed twice prior to acquisition on a FACSCanto II Immunocytometry system (BD Biosciences) and the data were analysed using FlowJo software version 10 (Ashland, OR, USA).

### Statistical analysis

Statistical analysis were performed using the non-parametric Kruskal–Wallis test using GraphPad Prism software version 7. Differences were considered statistically significant at a *P* value of <0.05.

## RESULTS

**Fig. 1.**
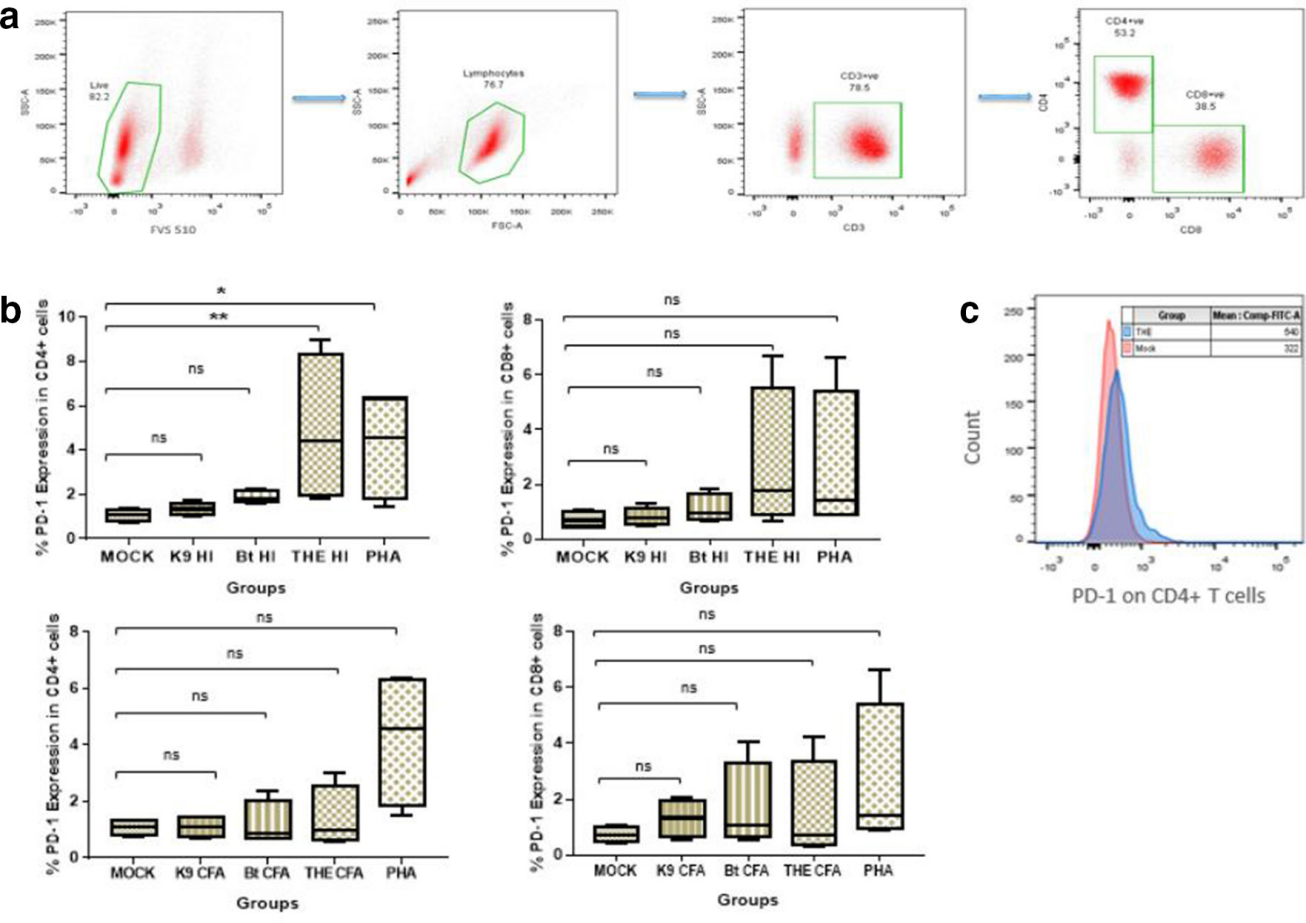
PD-1 expression upon exposure to HI bacteria. Peripheral blood mononuclear cells from healthy controls were stimulated *in vitro* with HI whole bacteria at an m.o.i. f 1 : 10 from the strains THE, *B. thailandensis* and K96243. (a) Gating strategy used for identifying T cell subsets. All gates were set using appropriate isotype controls. (b) PD-1 expression in CD4+ and CD8+ T cells following 18 h incubation with HI bacteria and culture filtrate antigen. Statistical analysis was performed using the Kruskal–Wallis test. P*<0.0125, P**<0.0025, P***<0.00025 with four Bonferroni comparisons. (c) An overlay histogram plot comparing mean fluorescence intensities of PD-1 in HI THE exposed and antigen-unexposed mock PBMCs. The data presented are representative of four individual experiments (*n*=4).

**Fig. 2.**
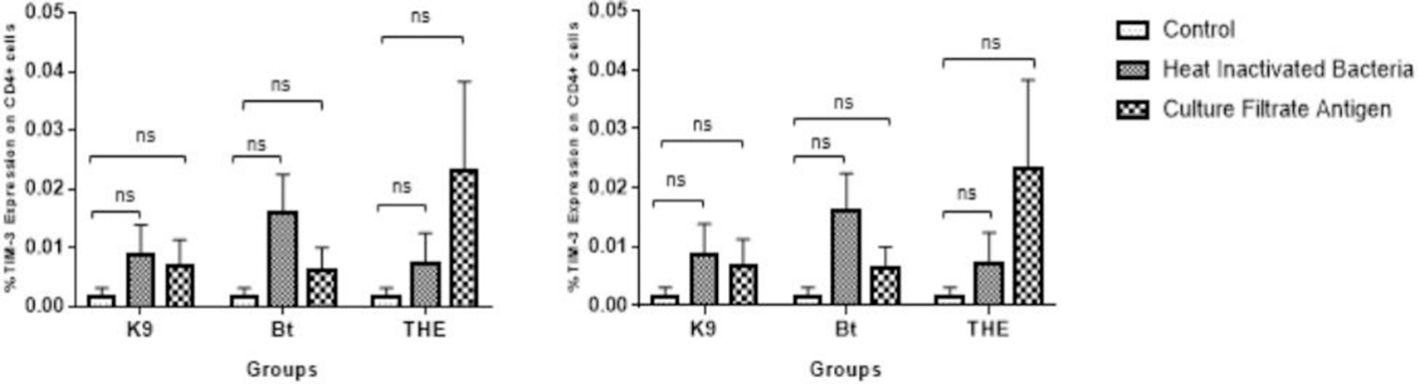
TIM-3 expression in CD4+ and CD8+ T cells following 18 h of exposure to HI bacteria and culture filtrate antigens. Peripheral blood mononuclear cells from healthy controls were stimulated *in vitro* with crude culture filtrates (10 ug ml^−1^) and HI whole bacteria at an m.o.i. of 1 : 10 from the strains THE, *B. thailandensis* and K96243. Statistical analysis was performed using the Kruskal–Wallis test. P*<0.0125, P**<0.0025, P***<0.00025 with four Bonferroni comparisons. The data presented are representative of four individual experiments (*n*=4).

PD-1 was recently shown to be upregulated in persistent *B. pseudomallei* infections in mice [36], which nonetheless has not been proven in humans. Here, healthy PBMCs were exposed to *B. pseudomallei* and *B. thailandensis* antigens *in vitro* and, following 18 h of incubation, cells were assessed for PD-1 and TIM-3 expression. We found that PD-1 was significantly upregulated upon exposure to HI *B. pseudomallei* strain THE compared to control, whereas *B. pseudomallei* strain K9 and *B. thailandensis* did not alter PD-1 expression. Interestingly, only CD4+ T cell subsets showed upregulated PD-1 upon exposure to HI THE strain compared to both control and *B. thailandensis* (Fig. 1). However, the CFAs did not alter PD-1 expression on both the T cell subsets (Fig. 1c). Next, we investigated the expression of TIM-3 on both the T cell subsets. There was no statistical significance in TIM-3 expression when compared to control (Fig. 2). We also observed the CD4+ and CD8+ T cell numbers and observed that there was no significant change upon exposure to *B. pseudomallei* and *B. thailandensis* antigens (data not shown).

## DISCUSSION

Recent research shows that the PD-1 pathway is emerging as an important mechanism exploited by many viruses and intracellular bacteria to dampen T cell responses [33–35]. We previously showed that PD-1 is highly upregulated during persistent *B. pseudomallei* infection in a murine model [36]. Although animal studies allow a closer approximation to human responses, we sought to validate if such PD-1 upregulation is translated in humans. We used PBMCs derived from healthy donors to provide a better understanding of immune responses during human melioidosis. The inclusion of the closely related non-virulent species *B. thailandensis* in our study helped to illustrate the prominence of PD-1 upregulation by virulent *B. pseudomallei *. Our findings showed that PD-1 is significantly upregulated by HI *B. pseudomallei in vitro*. The fact that PD-1 upregulation was seen in CD4+ T cells alone is contradictory to our recent reports on a mouse model, where both CD4+ and CD8+ T cells showed significant PD-1 upregulation [43]. However, previous studies have shown that CD4+ T cell proliferation alone was inhibited upon exposure to human PMNs pulsed with *B. pseudomallei* antigens [37]. We infer that the CD4+ phenotype of the T cells is important for host resistance against melioidosis in humans [37]. According to our results, only HI whole bacteria led to PD-1 upregulation and not culture filtrate antigen. *B. pseudomallei* is known to enter a viable but non-culturable (VBNC) state in response to environmental stress [44–46]. The incubation of *B. pseudomallei* at high temperatures for heat inactivation would have led them to enter a VBNC state and a possible resuscitation upon culturing with PBMCs would have allowed them to regain their ability to cause infection. However, the K96243 isolate did not cause any changes to PD-1 expression. It is unclear why these two strains of *B. pseudomallei* elicit varying degrees of T cell responses. The co-expression of PD-1 and TIM-3 has previously been reported in chronic viral infections [47, 48] and in *M. tuberculosis* infection [49, 50]. We investigated the possibility of PD-1 and TIM-3 co-expression in PBMCs exposed to *B. pseudomallei* and found that no T cells expressed TIM-3. Our study points to the likely role of PD-1 in regulating immune responses, especially those involving T cells in melioidosis, and supports the adoption of strategies to target PD-1 for developing newer therapeutic molecules for use in clinical treatment of melioidosis.
